# Toxicological Function of Adipose Tissue: Focus on Persistent Organic Pollutants

**DOI:** 10.1289/ehp.1205485

**Published:** 2012-12-05

**Authors:** Michele La Merrill, Claude Emond, Min Ji Kim, Jean-Philippe Antignac, Bruno Le Bizec, Karine Clément, Linda S. Birnbaum, Robert Barouki

**Affiliations:** 1 Department of Preventive Medicine, Mount Sinai School of Medicine, New York, New York, USA; 2 BioSimulation Consulting Inc., Newark, Delaware, USA; 3 Département de santé environnementale et santé au travail, Université de Montréal, Montréal, Québec, Canada; 4 INSERM UMR-S 747, Paris, France; 5 Université Paris Descartes, Centre Universitaire des Saints-Pères, Paris, France; 6 Assistance Publique-Hôpitaux de Paris, Hôpital Necker-Enfants Malades, Paris, France; 7 Université Paris 13, Sorbonne Paris Cité, INSERM U698, Bobigny, France; 8 ONIRIS, USC 2013 INRA, LABERCA, Atlanpole-La Chantrerie, Nantes, France; 9 INSERM, U872, Nutriomique équipe 7, Paris, France; 10 Centre de Recherche des Cordeliers, Université Pierre et Marie Curie-Paris 6, UMR S 872, Paris, France; 11 Assistance Publique-Hôpitaux de Paris, Hôpital Pitié-Salpêtrière, Département Nutrition et Endocrinologie, Paris, France; 12 CRNH-Ile de France, Paris, France; 13 National Cancer Institute, and; 14 National Institute of Environmental Health Sciences, National Institutes of Health, Department of Health and Human Services, Research Triangle Park, North Carolina, USA

**Keywords:** adipose tissue, aryl hydrocarbon receptor, development, diabetes, dioxin, inflammation, obesity, obesogens, polychlorinated biphenyls, toxicity, toxicokinetics

## Abstract

Background: Adipose tissue (AT) is involved in several physiological functions, including metabolic regulation, energy storage, and endocrine functions.

Objectives: In this review we examined the evidence that an additional function of AT is to modulate persistent organic pollutant (POP) toxicity through several mechanisms.

Methods: We reviewed the literature on the interaction of AT with POPs to provide a comprehensive model for this additional function of AT.

Discussion: As a storage compartment for lipophilic POPs, AT plays a critical role in the toxicokinetics of a variety of drugs and pollutants, in particular, POPs. By sequestering POPs, AT can protect other organs and tissues from POPs overload. However, this protective function could prove to be a threat in the long run. The accumulation of lipophilic POPs will increase total body burden. These accumulated POPs are slowly released into the bloodstream, and more so during weight loss. Thus, AT constitutes a continual source of internal exposure to POPs. In addition to its buffering function, AT is also a target of POPs and may mediate part of their metabolic effects. This is particularly relevant because many POPs induce obesogenic effects that may lead to quantitative and qualitative alterations of AT. Some POPs also induce a proinflammatory state in AT, which may lead to detrimental metabolic effects.

Conclusion: AT appears to play diverse functions both as a modulator and as a target of POPs toxicity.

Obesity is increasing in developed countries and is a commonly known risk for disorders such as impaired glucose tolerance, metabolic syndrome, diabetes mellitus, liver and cardiovascular disease (CVD), and cancer (Ludescher et al. 2009). Adipose tissue (AT) has historically been considered a simple storage tissue; however, its physiological functions have been appreciably reassessed over the last decade (Lafontan 2012), and evidence for metabolic and endocrine functions of AT has accumulated. More is known about the histological architecture of AT and the role of AT stroma, including immune cells. The pathological contribution of AT to obesity and metabolic disorders such as type 2 diabetes is gaining more attention. Recently, various interactions between AT and persistent organic pollutants (POPs) have been reported, suggesting that this tissue plays a significant role in the kinetics and the toxicity of POPs (Kim et al. 2011, 2012).

On the basis of these studies, we propose that AT, in addition to its other metabolic and endocrine functions, has diverse toxicological functions: *a*) AT can store a variety of hydrophobic xenobiotic chemicals, in particular POPs; *b*) AT constitutes a low-grade internal source of stored POPs leading to continuous exposure of other tissues; and *c*) AT can be a target for the effects of a xenobiotic chemical that alters AT functions, increases AT inflammation, and/or modulates the differentiation of AT precursor cells. For instance, obesogens are exogenous chemicals (from a nutritional, pharmaceutical, or environmental origin) that directly or indirectly increase obesity through disruption of metabolic, hormonal, or developmental processes (Grun and Blumberg 2007; Schug et al. 2011). Conversely, several POPs are known to induce cachexia, particularly at high doses. In this review, we discuss these issues and present evidence that supports a complex, previously unsuspected role of AT in toxicology.

## Metabolic and Endocrine Functions of AT

AT is classically viewed as the main reservoir of energy mobilized from the body. In fact, AT is not merely an energy depot, it is essential for normal carbohydrate and lipid homeostasis. When stimulated by insulin, adipocytes store glucose as triglycerides in lipid droplets (Stolic et al. 2002). Adipocytes meet the energy needs in states of metabolic stress, such as fasting, by releasing fatty acids through lipolytic processes (Lafontan 2012). In addition to the energy-storing function of AT, adipocytes secrete several endocrine factors such as leptin and adiponectin, which regulate appetite as well as metabolic and inflammatory functions (reviewed by Galic et al. 2010). AT has substantial functional breadth in part because of the great diversity of cells within this tissue, such as adipocyte precursors (preadipocytes) in different states of differentiation, vascular cells, central nervous system cells, fibroblasts, and immune cells. In addition to adipocytes, AT is a site of storage and production of various substances with autocrine, paracrine, and neuroendocrine actions that influence behavior, energy regulation, lipid oxidation, immune and vascular function, and hormonal status, as well as its own metabolism and cellularity (reviewed by Galic et al. 2010; Ouchi et al. 2011).

Obesity is characterized by adipocyte hypertrophy but also by the accumulation of macrophages in AT depots. Accumulation of macrophages in the visceral AT depot, but not the subcutaneous depot, is associated with liver injury (Tordjman et al. 2012). *In vitro* experiments have shown that macrophage secretions profoundly perturb adipose cell biology, promoting a proliferative, proinflammatory, and profibrotic state of preadipocytes, as well as an insulin-resistant state of mature adipocytes (Dalmas et al. 2011). Lymphocytes, natural killer cells, and mast cells are found in AT parenchyma in obese people, and also in fibrosis depots that accumulate in obese subjects (Divoux et al. 2010).

AT is much more than just an energy storehouse for the body or a repository for lipophilic chemicals. Obesity affects not only AT structure but its function. Thus, because of these critical AT functions, the interaction of POPs with AT could lead to substantial metabolic and endocrine disruption.

## AT as a Mechanism of Protection

*Evidence of a protective function of AT.* One of the most critical survival functions in a complex chemical environment is the ability of cells and organisms to detoxify and eliminate xenobiotic chemicals. The best studied detoxification machinery is the xenobiotic metabolizing system, which includes receptors, metabolizing enzymes, and transporters, and which tends to prevent absorption, increase water solubility, or decrease reactivity of xenobiotic chemicals, thus leading to their detoxification and elimination from the body (Barouki 2010). However, POPs are an important class of xenobiotic chemicals that are resistant to metabolism. POPs are environmentally and biologically persistent, which leads to their bioaccumulation and biomagnification up the food chain. Fatty foods of animal origin (e.g., meat, fish, dairy) are important vectors of several classes of POPs, including dioxins and polychlorinated biphenyls (PCBs) (Bergkvist et al. 2008). POPs include certain organochlorine pesticides; polyhalogenated dibenzo-*p*-dioxins, furans, and biphenyls; and certain polybrominated flame retardants and perfluorinated chemicals. POPs do not readily undergo degradation by xenobiotic metabolizing enzymes, largely because of their high degrees of halogenation. However, some POPs bind—often with high affinity—to certain xenobiotic receptors, as well as to certain xenobiotic metabolizing enzymes such as CYP1A2 (cytochrome P450 1A2), without undergoing catalytic transformation (Diliberto et al. 1999). Such binding plays a significant role in the distribution of POPs. Because of their hydrophobicity, POPs tend to distribute into lipophilic compartments, particularly the AT.

POPs are taken up by adipocytes and localize within lipid droplets (Bourez et al. 2012). However, the precise location of POPs within AT and their actual effects at the subcellular level are poorly understood. The accumulation of POPs within AT is believed to decrease their availability to other cells and tissues, thereby limiting their systemic toxicity. Experimental evidence supports such a protective function for AT. Studies conducted in the 1980s and 1990s showed an inverse correlation between POPs toxicity and fat mass of different animal species. Geyer et al. (1993) compared the 30-day toxicity of 2,3,7,8-tetrachlorodibenzo-*p*-dioxin (TCDD) in approximately 20 terrestrial animal species and found a positive correlation between the adiposity of these species and their median lethal dose. These authors concluded that the species with the highest fat mass tended to display more resistance to TCDD in this acute toxicity test. Their conclusions were in line with studies showing that resistance of aquatic species to dioxin was also related to their fat mass content (Lassiter and Hallam 1990). However, these observations should not be taken as evidence that adiposity is the only factor discriminating dioxin-sensitive and -resistant species. There is clear genetic evidence for a major contribution of the aryl hydrocarbon receptor (AhR) to dioxin toxicity.

*The toxicokinetic role of AT.* AT plays a major role in the storage and toxicokinetics of POPs because of their lipophilicity. The histological and anatomical structure of different types of AT can influence their contribution to toxicokinetics. Recently, Sbarbati et al. (2010) proposed a new AT classification based on AT organization, structure, surrounding tissue, and anatomical localization. Additional studies are needed to determine whether different properties of these AT subtypes could have a pharmacokinetic impact on POPs. However, there is currently no evidence for differences in POP content under steady-state conditions between different types of AT (Kim et al. 2011).

Despite the presence of a large number of AT cell types, POP storage in AT is believed to be primarily in the adipocytes (Bourez et al. 2012). Adipocyte cytoplasm is almost totally composed of triglyceride droplets (Sbarbati et al. 2010). The transfer of POPs from the vascular environment into the cell or through other tissue structures implies that pharmacokinetic factors such as tissue volume, anatomical localization, and blood flow rate influence the distribution of the chemicals into AT. The default approach is to assume that the tissue is flow limited, which means that the distribution of chemicals contained in blood across the well-stirred tissue compartment is fast and homogenous. Although this assumption is valid for the distribution of many xenobiotic chemicals into many tissues or organs, it appears to be incorrect for the movement of several highly lipophilic POPs across the AT because of their diffusion-limited (also called permeability limited) characteristics (Levitt 2010). In this case, the distribution of the chemicals is slower and may be incomplete. The physical basis of this AT diffusion limitation is related to the octanol:water partition coefficient (*K*_ow_). The diffusion limitation is related to the exchange rate between the blood and adipose lipid, which becomes rate limiting if *K*_ow_ is large enough (Levitt 2010). In addition, the diffusion limitation values take into account the thickness and diameter of the adipose capillary network, as well as diffusion across the membrane. At steady state, the log *K*_ow_ predicts the capacity of the chemical to diffuse into AT and accumulate.

The best prospective mathematical pharmacokinetic method to estimate diffusion coefficients in AT is physiologically based pharmacokinetic (PBPK) modeling (Emond et al. 2006). Using PBPK modeling, we assumed that the diffusion coefficient parameter was constant across AT for a rate ranging between 4.5% and 5.0% of the cardiac output of 5.60 L/min (Derelanko and Hollinger 1995). However, as previously described by Levitt (2010), the rate of diffusion in AT is usually lower than that, resulting in a delay to reach steady state between blood and AT. In the future, we may need to determine these parameters in different AT types because there is some evidence that the rate of diffusion may vary in different depots.

Another important issue, especially for obese people, is that the classical pharmacokinetic analysis may lead to error in the estimate of distribution volume during steady state (*V*_ss_). Using classical pharmacokinetic calculations to model highly lipophilic POPs at low concentrations often leads to a substantial underestimation of *V*_ss_ and mean residence time during the late terminal phase of the elimination time curve. An accurate determination of *V*_ss_ is required in sound clinical practice because it is critical for the proper selection of a drug treatment regimen or of environmental chemical distribution and kinetics (Berezhkovskiy 2011). Several laboratories use magnetic resonance imaging to more accurately study the apparent diffusion coefficient, the distribution of AT in the body, the volume of AT in the different regions, and the different rates of diffusion (Steidle et al. 2011).

When considering POPs as obesogens, it is valuable to revisit the evidence pertaining to their lipophilicity in various tissues. TCDD and dichlorodiphenyltrichloroethane (DDT) are transported out of the gut in the triglyceride phase of chylomicrons (Vost and Maclean 1984). DDT and its metabolites also conjugate to hepatic fatty acids, including stearic, oleic, linoleic, and palmitic acids (Leighty et al. 1980). Although several PCBs and organochlorine pesticides in blood are associated with the protein fraction, they are also associated with all major lipoprotein compartments [very low density lipoprotein (VLDL), low density lipoprotein (LDL) high density lipoprotein (HDL)] (Vost and Maclean 1984). TCDD was also found in the same lipoprotein compartments of apolipoprotein E–deficient (ApoE^–/–^) and wild-type mice (Dalton et al. 2001). Although these POP–lipid associations are considered responsible for their tissue partitioning, they may also be partially responsible for POP lipotoxicity (Leighty et al. 1980).

Recent studies have suggested that some heterogeneity exists with respect to both the distribution of POPs across AT depots (Ronn et al. 2011; Roos et al. 2012) and the association of individual POPs with AT mass (Ronn et al. 2011; Roos et al. 2012; Yu et al. 2011). However, studies of the heterogeneity of POP distribution across AT depots need to be confirmed because of the limited number of subjects that were studied (Ronn et al. 2011; Roos et al. 2012). Some differences in the association of individual POPs with AT mass may be explained by differential lipophilicity of various POPs. For instance, circulating levels of highly chlorinated PCBs have a negative association with AT mass (e.g., PCBs 153, 156, 157, 169, 170, 180, 189, 194, 206, and 209), whereas circulating levels of relatively low-chlorine–containing PCBs have a positive association with AT mass (e.g., PCBs 74, 99, 105, and 118 (Ronn et al. 2011; Roos et al. 2012; Yu et al. 2011). Confirmation of the importance of this heterogeneity may contribute to a better understanding of the relationship between the POP profiles in both serum and AT. The utility of an environmental contamination signature for the evaluation of food contamination needs further assessment in humans (Antignac et al. 2006).

Clearly, toxicokinetics and computational biology represent important approaches that are needed to understand the interaction of chemicals and AT. Using recent technical advances, a more quantitative and accurate assessment of these interactions will be possible in the future.

## AT as a Source of Chronic POP Exposure

As mentioned above, POPs and other lipophilic xenobiotic chemicals distribute according to their affinity for proteins and lipids and are stored primarily in the AT. They are also found in blood, from which they can contaminate other tissues. Several observations in both humans and animals suggest that the release of pollutants from AT is an important source of blood POPs.

In humans, most of the evidence has been gathered from studies on drastic weight loss in obese individuals. Such weight loss can be achieved through dietary changes and bariatric surgery and could lead to a decrease exceeding 30 kg of fat mass. Several independent studies have shown an increase in blood POPs following fat loss elicited by dietary changes either alone or coupled with bariatric surgery (Hue et al. 2006; Kim et al. 2011). The role of fat mass in the control of POP blood levels was further supported by Lim et al. (2010) who demonstrated an inverse correlation between long-term weight changes and POP serum concentrations.

If increased blood levels of POPs during weight loss are related to their release from AT, changes in AT POP content would be expected. This has been addressed by Kim et al. (2011) who determined POP concentrations in both blood and AT and also assessed the total amount of fat in the studied individuals. Their data indicated that the POP concentration in AT (expressed per gram lipid) increased with weight loss. Although this may seem paradoxical, it is not surprising because the total amount of fat mass decreases considerably, thereby leading to an increased concentration of pollutants in AT. Released POPs can be taken up readily by the remaining fat, which is essentially an infinite sink. Nevertheless, this total POP body burden tends to decrease by 15% after weight loss, at least for certain POPs (Kim et al. 2011). The primary excretion route of most POPs is feces, but routes may also include maternofetal transfer and lactation (Wendling et al. 1990).

Evidence from wildlife indicates that fasting and AT loss increase circulating POPs. Debier et al. (2006) conducted observational studies in northern elephant seals, which accumulate a large amount of fat to cope with fasting that could last several weeks and result in a large amount of AT loss. The authors showed that fasting was accompanied by an increase in the serum concentration of PCBs, likely due to the release of PCBs from contaminated fat depots. Interestingly, the concentration of PCBs also increased in blubber because of the decrease in body fat mass. Thus, the mobilization of POPs during fasting may lead to toxic effects.

Experimental evidence also suggests that fasting results in redistribution of POPs from their AT storage sites. Indeed, Jandacek et al. (2005) observed that in rodents pretreated with hexachlorobenzene, weight loss led to a time-dependent increase in the brain content of hexachlorobenzene. In a study in which mice were pretreated with DDT, weight loss led to increased DDT in all tissues examined (e.g., brain, lung, heart, spleen, kidney, liver, adipose tissue, blood) except muscle (Ohmiya and Nakai 1977). However, there was no evidence of a change in DDT metabolism or excretion. Thus, decreased AT appears to lead to a redistribution of certain POPs, which favors movement of POPs toward other lipid-rich tissues. The enhanced localization of DDT in the brain was associated with toxic central nervous system outcomes (Ohmiya and Nakai 1977).

A critical issue is whether the release of POPs from AT during weight loss could also lead to toxic outcomes in other organs and tissues of humans. Indirect evidence was obtained in humans from several studies of weight loss triggered by either diet or diet associated with bariatric surgery. Tremblay and colleagues (Imbeault et al. 2002; Pelletier et al. 2002; Tremblay and Chaput 2009) showed that increased serum POPs correlated with alterations in resting metabolic rates, thermogenesis, and oxidative capacity of skeletal muscle. In a study by Kim et al. (2011), all individuals undergoing weight loss had improved blood lipid and liver toxicity parameters, but those who had the highest serum POP levels showed a delay in improvement of these parameters. This suggests that POPs may counteract the positive effects of weight loss on hepatic and serum lipids.

The concentration of POPs in breast milk reflects the POP body burden in an individual; indirect evidence for POP release from fat storage tissue in humans has been provided by breastfeeding studies. Many POPs and other xenobiotic chemicals are found in breast milk because of its lipid content. Because of the equilibrium between lipid-associated POPs in AT, blood, and milk, it is likely that a significant fraction of breast milk POPs originates from the AT storage compartment, as well as from newly absorbed contaminants. In agreement with this model, in a study of female rats, You et al. (1999) observed AT DDE concentrations that were two to three times greater at the end of gestation than after weaning of offspring. Milbrath et al. (2009) reported that the apparent half-life of dioxin in humans is reduced by breastfeeding. While considering the potential negative consequences of POP presence in breast milk, one should keep in mind the important nutritional and immune benefits of breastfeeding.

**Figure 1:**
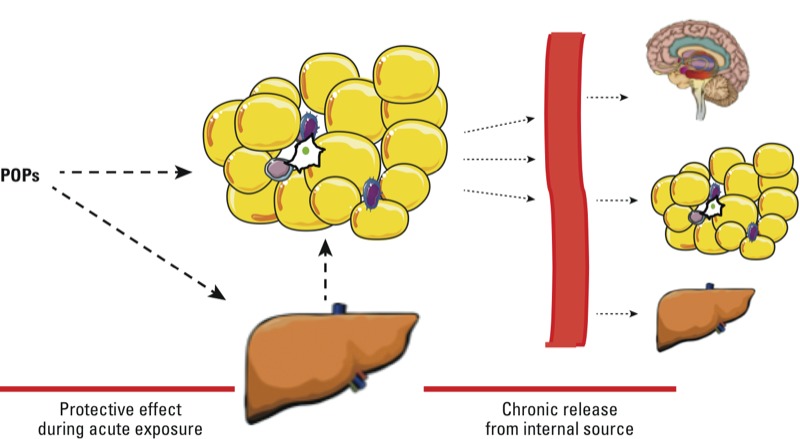
Dual role of AT in the regulation of POP kinetics. Upon exposure to POPs, these lipophilic pollutants are stored in liver and AT (left); this prevents the action of these pollutants in other sensitive tissues and may be protective to a certain extent. POPs released from their storage site in AT constitute a source of low-level internal exposure (right).

Several human and animal studies have suggested that AT behaves as a toxicokinetic buffer for lipophilic pollutants (Figure 1). AT is a specific storage compartment for these pollutants. However, this is a dynamic situation, and release from AT occurs at a low basal level that can be magnified during weight loss. There is indirect evidence suggesting that released POPs exert some toxic effects (Kim et al. 2011); however, more direct evidence is needed.

## AT as a Target of Pollutants

**Figure 2:**
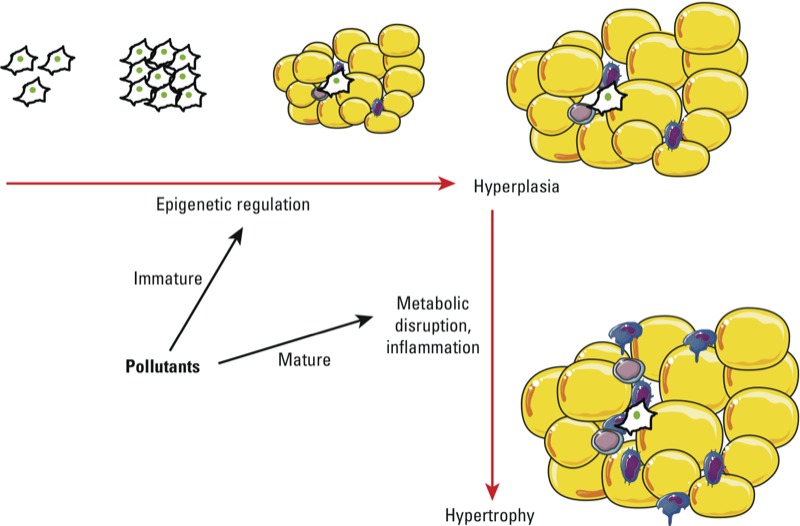
POPs as obesogens and as disruptors of AT structure and function. Strong evidence from both *in vivo* and *in vitro* studies suggests that POPs can influence the development of AT, particularly at low doses. These programming events take place in early life (e.g., fetal, neonatal), probably through epigenetic mechanisms, and could have an impact on diseases in adulthood. In addition, POPs can alter AT function and structure later in life; this occurs primarily through metabolic disruption and inflammation. These effects favor the development of metabolic diseases.

*POPs as obesogens.* With the study of obesogens still in its infancy, experimental research on POPs as obesogens is sparse. In a recent review of the literature on developmental exposures that increase risk of obesity, with an emphasis on human exposures, we found that themes are already emerging (La Merrill and Birnbaum 2011). Development (e.g., prenatal, postnatal, pubertal) is likely a critical window of susceptibility to obesogen effects of toxic exposures (Figure 2). Programming mechanisms are still unclear but are believed to involve epigenetic regulation of critical genes that lead to adiposity later in life (Barouki et al. 2012). Evidence suggests that developmental exposures to chemicals that increase the risk of obesity sometimes operate in a nonmonotonic dose–response manner; cachexia may occur at high doses, whereas body and/or adipose mass gain occurs at low doses of the same chemical. Further, there may be sex-specific effects of developmental toxic exposures that increase the risk of obesity (Tang-Peronard et al. 2011). Here, we focus on experimental research on POPs that cause obesity and dyslipidemia. Developmental exposures to these same POPs are positively associated with obesity in humans (Valvi et al. 2012).

Some rodent models have indicated that dioxin-like (DL) chemicals may be obesogens. Zhu et al. (2008) reported that body weights of adult C3H/HeN mice exposed to 100 µg TCDD/kg body weight once every 2 weeks for 8 weeks were > 40% higher than those of control mice. This body weight change was seen only when mice were fed a high fat diet, which was not out of the range of an American diet. In a 1-month study, chronic developmental exposure to the PCB mixture Aroclor 1254 was associated with increased body weights of mouse pups on postnatal days (PNDs) 16–20 (Branchi et al. 2002). In another study, adult mice exposed to 49 mg DL PCB-77/kg body weight had an AhR-dependent increase in body mass (Arsenescu et al. 2008). Hennig et al. (2005) reported that the same dose of PCB-77 increased body mass, fatty liver, abdominal fat, and adipocyte hypertrophy in CVD mice. Fatty liver, attributed to increased hepatic triglycerides and cholesterol, was also observed in mice treated with 50 mg DL PCB-169/kg body weight (Kohli et al. 1979).

There is limited evidence of increased adiposity in animal studies of non-DL POPs; however, body fat is seldom assessed in studies reporting no increased body mass after POP exposure (La Merrill and Birnbaum 2011). Prenatal exposure to a major polybrominated diethyl ether (BDE-99; 2,2´,4,4´,5-penta-BDE) increased mouse birth weight (Lilienthal et al. 2006), and prenatal and postnatal exposure to BDE-47 (2,2´,4,4´-tetra-BDE) increased rat body weights from birth to puberty (when the study ended) (Suvorov et al. 2009). In the longest study of developmental PBDE exposure to examine body weights, Gee and Moser (2008) found that male mice exposed to BDE-47 10 days after birth had increased body weights from PND47 until the end of the study, when animals were 4 months of age. These studies all indicate significant effects in body composition after perinatal exposure to PBDEs; however, the mechanisms remain unclear and the data should be interpreted with caution because certain preparations of BDEs could be contaminated with DL chemicals. After perinatal exposure to perfluorooctanoic acid (PFOA), obesogenic effects do not appear until later in life. Mature mice that were exposed to low levels of PFOA *in utero* had increased body mass, with an inverted U-shaped dose–response curve (Hines et al. 2009). When mice reached 18 months of age, there was no longer an effect on mouse weight; however, the authors observed a positive dose–response relationship between *in utero* PFOA exposure levels and abdominal brown AT mass but a negative relationship between PFOA and white AT mass. Consistent with experimental findings, a recent prospective human study demonstrated that maternal PFOA levels during pregnancy were associated with obesity in the daughters 20 years later (Halldorsson et al. 2012). Organochlorine pesticides may also increase adiposity. For example, Rivett et al. (1978) reported that low doses of lindane elevated the body weights of dogs. Likewise, oral DDT exposure increased the body weights of female mice and their offspring in a two-generation chronic-exposure study (Tomatis et al. 1972).

*Evidence and implications of lipotoxicity.* The accumulation of lipids in non-adipose tissues has toxic effects on tissue function, and this lipotoxicity may lead to diabetes, hypertension, and heart disease. Many of the POPs that associate with lipids disrupt their homeostasis. Lipotoxicity and dyslipidemia induced by dioxin and DL PCBs occur even in the absence of an obese phenotype. For example, PCB-77 has been shown to elevate serum VLDL in ApoE^–/–^ mice (Arsenescu et al. 2008; Dalton et al. 2001). Similarly, TCDD caused an AhR-dependent increase in the cholesterol content of atherosclerotic plaques and elevated serum LDL in ApoE^–/–^ mice (Arsenescu et al. 2008; Dalton et al. 2001; Wu et al. 2011). The AhR appears to have an innate role in lipid homeostasis. The AhR is activated by LDL, and AhR-knockout mice have higher levels of serum LDL (McMillan and Bradfield 2007). Further, AhR knockout *Caenorhabditis elegans* larva have elevated fatty acids (Aarnio et al. 2010). There is also evidence to suggest that activity of the AhR nuclear translocator (ARNT; which forms a heterodimer with AhR) is required for lipogenesis and glycolysis (Pillai et al. 2011; Wang et al. 2009).

Experimental organochlorine pesticide exposures cause systemic lipotoxicity. DDT exposure increased cholesterol and triglycerides in both serum and AT (Sanyal et al. 1982), and increased hepatic triglyceride synthesis (Sanyal et al. 1982). Similarly, increased triglyceride synthesis was observed in dieldrin-exposed rats (Bhatia and Venkitasubramanian 1972). Hepatic fatty acid composition and utilization was also altered when DDT, endosulfan, or dieldrin were administered to rats (Kohli et al. 1975; Narayan et al. 1990).

Lipotoxicity has also been observed after exposure to brominated flame retardants and perfluorinated chemicals. Both male and female rats exposed to a commercial penta-BDE mixture exhibited a dose-related increase in plasma cholesterol (van der Ven et al. 2008). In another study of rats exposed to a commercial penta-BDE mixture, lipolysis rates were increased in *ex vivo* adipocytes (Hoppe and Carey 2007). However, low doses of PFOA reduced total cholesterol and triglycerides in adult rats; however, in mice, low-dose PFOA had no effect on cholesterol but increased triglycerides (Loveless et al. 2006). Although the lipid-lowering effect of PFOA exposure in these rodent studies is consistent with peroxisome proliferator-activated receptor α (PPARα) agonism (Klaunig et al. 2003), PFOA is consistently associated with elevated cholesterol in humans (Steenland et al. 2010). Results of PFOA and perfluorooctane sulfonate (PFOS) exposures in PPARα knock-out mice have shown changes in gene expression indicative of lipotoxicity (Rosen et al. 2008; Rosen et al. 2010) and altered fatty acid metabolism (Rosen et al. 2008). Similarly, PFOS-exposed mice had altered gene expression associated with lipid metabolism (Rosen et al. 2010).

These experimental findings have important implications for epidemiology studies: POP levels in a given tissue are often normalized to lipid content of that tissue. By assuming that the total body burden of POPs is evenly distributed in all lipid stores, POP concentrations in different matrices can be easily compared. Unfortunately, the assumption of even distribution of POPs is not always valid. In addition, the correlation and attributable variation of POPs to lipids varies across studies (Guo et al. 1987; Porta et al. 2009), which is partially due to variation in the lipid extraction methods used by investigators. If some POPs cause both obesity and dyslipidemia through a common causal pathway, normalizing POPs to lipids may inadvertently adjust the effect of POPs toward the null. Indeed, in a recent longitudinal epidemiology study, Lee et al. (2011) found weaker, but still significant, associations between POPs and obesity when adjusting for serum triglycerides and cholesterol; this suggests that analysis of lipid-adjusted POPs may represent an overadjustment, given that these chemicals may also perturb lipid metabolism. In the absence of definitive information about the causal pathway of the effects of POPs on outcomes for which dyslipidemia may be on the causal pathway (including obesity, diabetes, CVD, and the cancers for which obesity and diabetes increase risk, such as breast cancer), we recommend presenting analyses of POPs both with and without lipid adjustment, which is supported by other scientists (Porta et al. 2009).

*Disruption of AT function and adipocyte differentiation.* The mechanisms through which POPs could induce the disruption of AT function, metabolism, and adipose cell differentiation are diverse. Here we present only a few of these issues. Results of *in vitro* studies are consistent with a positive role of POPs in the risk of obesity. In addition, POPs can act by altering the activity of metabolic enzymes. For example, both TCDD and PCB-77 have been reported to reduce lipoprotein lipase (LPL) activity *in vitro* unless AhR antagonists were present (Hegele 2009; Olsen et al. 1998). *LPL* mutations are associated with severe hyperlipidemia in humans.

POPs can also alter adipocyte differentiation; however, the literature in this field is somewhat contradictory. Arsenescu et al. (2008) observed that low doses of TCDD and PCB-77 could induce adipocyte differentiation *in vitro*, with greater potency of PCB-77 than its toxic equivalency factor (TEF) would suggest. In different studies, overexpression of the AhR was shown to decrease adipocyte differentiation and expression of PPARγ, a marker of adipocyte terminal differentiation (Cho et al. 2005; Tontonoz and Spiegelman 2008). Additional evidence suggests multiple and often antagonistic interactions between the AhR and the PPARγ pathways (Remillard and Bunce 2002). Other mechanisms have also been suggested to account for the effect of DL chemicals on adipocyte differentiation; these include the interaction with hormonal or retinoic acid receptors or through the regulation of CCAAT/enhancer binding protein (C/EBP) protein family isoforms (Mullerova and Kopecky 2007; Vogel et al. 2004). In certain cellular systems, large-scale studies suggested cooperative antiadipogenic effects of dioxin and growth factors (Hanlon et al. 2005). It is likely that some of the apparently contradictory data are due to different cellular systems, different developmental stages, different species, and different xenobiotic chemical doses. For example, DL chemicals may promote adipocyte differentiation at low doses and display an opposite effect at higher doses.

Cellular and animal studies examining other POPs also indicate pro-obesogenic effects. PPARγ agonism is commonly associated with most candidate obesogens, including perfluoroalkyls, DDT, organotin, phthalates, and thiazolidinediones (Kopec et al. 2010; La Merrill and Birnbaum 2011). For instance, DDT is capable of inducing dose-dependent adipocyte differentiation through increased PPARγ expression (Moreno-Aliaga and Matsumura 2002). These mechanistic studies suggest complex, multiple, and dose-dependent effects of POPs on AT differentiation. Whereas the AhR pathway is clearly implicated, other pathways are also involved, leading to nonmonotonic dose–effect relationships. Future research should clarify these complex and sometimes contradictory effects.

Because of the importance of the inflammatory phenotype in metabolic diseases, one possible action of POPs would be induction of AT inflammation. Many POPs are well-characterized immunotoxicants. Several studies have shown that POPs increase the expression of inflammatory genes in adipose cells (Arsenescu et al. 2008; Kern et al. 2002; Li et al. 2008). We have recently shown, in a human model of preadipocytes and adipocytes, that the primary effect of TCDD on gene expression was the induction of the inflammatory pathway (Kim et al. 2012). Furthermore, treatment of mice with 10 µg/kg body weight of TCDD led to increased gene expression of several cytokines as well as other inflammatory mediators in AT, and, importantly, increased the number of macrophages in this tissue (Kim et al. 2012). Interestingly, in obese individuals, increased AT inflammation correlates with increased metabolic disruption such as insulin resistance and diabetes. These observations suggest that, in addition to their effects on obesity, POPs may contribute to AT inflammation, thereby increasing the likelihood of metabolic disruption (Figure 2).

**Figure 3:**
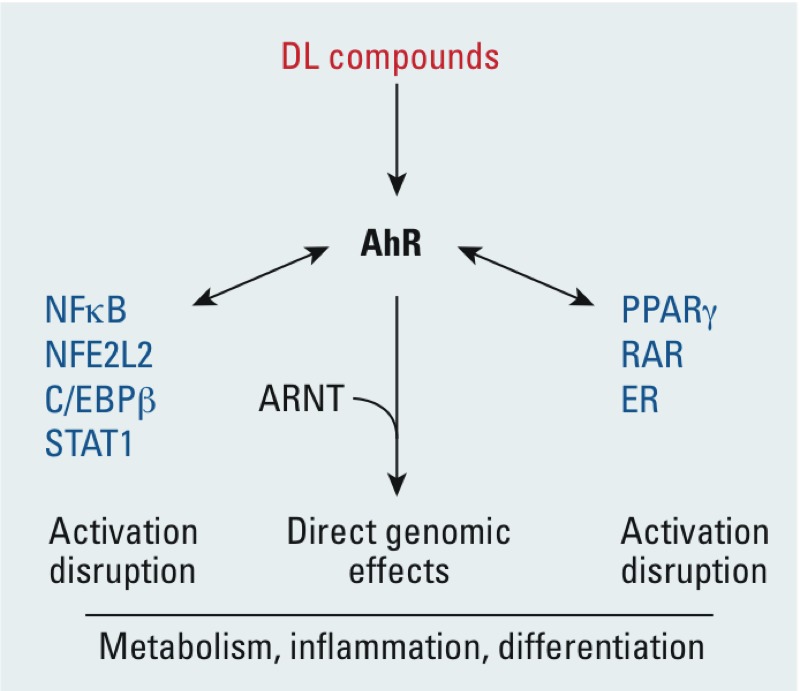
Major signaling mechanisms involved in the effects of DL POPs on AT. Abbreviations: ER, estrogen receptor; RAR, retinoic acid receptor. Most, if not all, of the effects of DL compounds are mediated by the AhR. Only genomic effects are shown. The AhR could directly regulate target genes as a heterodimer with ARNT. Several interactions with transcription factors or nuclear receptors that have been described are shown here. POPs could either trigger these interactions or disrupt existing interactions between the AhR and other signaling factors.

The mechanisms of DL chemical regulation of inflammation are complex and may depend on the system that is studied. Both anti-inflammatory and proinflammatory effects have been described. Because of the endogenous role of the AhR in the regulation of immunity, exogenous AhR ligands could either mimic or disrupt these pathways thereby influencing the regulation of inflammatory gene expression (Esser et al. 2009) (Figure 3). In addition, complex interactions between the AhR and critical transcription factors are involved in the regulation of inflammation, such as nuclear factor of kappa light polypeptide gene enhancer in B cells (NFκB) (Tian 2009; Vondracek et al. 2011). These interactions can also be observed in the absence of xenobiotics. For instance, the AhR forms a complex with signal transducer and activator of transcription 1 (STAT1) and NFκB to negatively regulate the innate inflammatory response even in the absence of an exogenous ligand (Kimura et al. 2009) (Figure 3). The interactions of the AhR with nuclear factor, erythroid derived 2, like 2 (NFE2L2) signaling could also account for its regulation of inflammation both in adipocytes and in other cells (Haarmann-Stemmann et al. 2012; Shin et al. 2007). The AhR and its ligands clearly modulate the inflammatory response. These effects could be due to the perturbation of an endogenous function of this receptor, as well as to additional effects triggered by xenobiotic activation.

## Conclusion

The studies discussed here indicate that AT plays a central role in POP toxicology. This role is complex and may seem paradoxical. Indeed, there is evidence that AT is protective under conditions of acute or subacute exposure to POPs. Storage in the lipid droplets has a buffering effect and prevents the persistence of high blood levels of these POPs and also prevents high POP exposure of other more sensitive lipophilic tissues such as the brain.

Furthermore, it is presently unclear where and how POPs are stored within the lipid droplet, whether associations between POPs and lipids alter lipid dynamics, and whether adipocytes or other AT cells are exposed to higher concentrations of POPs because of increased residence time in tissues. If this longer residence time is responsible for higher POP concentrations in AT cells, the storage function of AT may lead to increased toxicity of POPs toward this tissue. If confirmed, this would indicate that the effects of POPs on metabolic diseases such as diabetes could be explained by primary toxicity to AT, including inflammation, disruption of metabolism, and altered differentiation. Another likely consequence of the POP-storage function of AT is that this tissue constitutes an internal source of low-level chronic exposure of the organism to POPs. This is best illustrated by studies of drastic weight loss in which a release of POPs into the bloodstream is associated with metabolic and liver toxicity (Kim et al. 2011).

There is ample evidence that AT is also a direct or indirect target of POP toxicity. The obesogen concept, which highlights the vulnerability of the fetal and childhood periods of life in which tissue and organ development take place, suggests that AT development could be a specific target of POP exposure (Barouki et al. 2012). It is unclear whether increased adiposity is a direct effect, as suggested by *in vitro* studies, or an indirect effect mediated by metabolic disruption elicited by certain POPs (Arsenescu et al. 2008; Kim et al. 2012). There are a number of possible explanations for the obesogenic effects of POPs. One possibility is that these effects may be linked to the storage function of AT and thus that increased adipose mass is a long-term adaptive response to POP exposure. It is also intriguing that both POP exposure and nutritional imbalance disrupt metabolic programming leading to obesity and metabolic diseases. Whether these have similar mechanisms is a matter for further study. POPs not only display quantitative effects on AT (increased fat mass) but they also alter AT quality, notably through inflammation (Figure 2). These alterations are known to increase the risk of obesity.

Several unanswered questions still need to be addressed.

Obesogens and epigenetics. The obesogen concept needs to be supported by relevant mechanisms of action. To date, epigenetic alterations appear to be the most likely mechanisms that could explain perinatal programming leading to later-life obesity and metabolic diseases (Barouki et al. 2012). Although several POPs have been shown to elicit modifications in DNA methylation or microRNA expression (Baccarelli et al. 2009; Manikkam et al. 2012; McClure et al. 2011), it is still unclear whether such alterations are directly implicated in the obesogenic effect. Research should primarily focus on these issues. It is also important to assess the effects of POPs on stem cells and to identify the most relevant *in vitro* systems. Clearly the validation of a predictive *in vitro* system to test putative obesogenic compounds is an important target for future research.

POP location and dynamics. Additional studies should assess the actual localization of POPs within the adipose cell and lipid droplet, as well as the dynamics of POPs following their storage in the AT and after weight loss. These studies should also account for heterogeneity of POP distribution that is dependent on variation both within and between the class of POPs as well as on their physiochemical properties.

Mechanisms of action. Experimental studies should attempt to identify the mechanisms involved in POP action on the AT. In many cases, these mechanisms are somewhat contradictory (e.g., both proinflammatory and anti-inflammatory effects). Understanding these issues is critical. They may be related to, for example, dose, cellular target, or physiological context. The presence of multiple mechanisms could explain nonmonotonic dose–response curves.

Endogenous functions. The possible involvement of the AhR, as well as other target receptors, in endogenous functions suggest that xenobiotic ligands may disrupt these endogenous functions and/or lead to additional toxic effects. Delineation of these effects *in vitro* and *in vivo* is critical to improve our knowledge in this field.

Human studies. Biomonitoring of POPs within clinical and epidemiological studies is critical to validate experimental observations reviewed here and to support public health action. Prospective longitudinal studies are the most useful tools in establishing causal relationships with POP exposures. In addition, investigations that include a more detailed characterization of exposures (especially perinatal exposures) are invaluable to help identify obesogens and metabolic disruptors.

Overall, AT appears to be a major player in the toxicological responses to POP exposure, both in terms of adaptation and toxic effects. We hope that the toxicological community will give further attention to this important tissue when examining the detrimental effects of pollutants and drugs.
